# Comparative evaluation of the effect of different growth media on in vitro sensitivity to azithromycin in multi-drug resistant *Pseudomonas aeruginosa* isolated from cystic fibrosis patients

**DOI:** 10.1186/s13756-020-00859-7

**Published:** 2020-12-09

**Authors:** Michael Sörensen, Bakhodur Khakimov, Dennis Nurjadi, Sébastien Boutin, Buqing Yi, Alexander H. Dalpke, Tatjana Eigenbrod

**Affiliations:** 1 Department of Infectious Diseases, Medical Microbiology and Hygiene, Heidelberg University Hospital, 69120 Heidelberg, Germany; 2 Laboratory Enders and Partners, 70193 Stuttgart, Germany; 3 Institute of Medical Microbiology and Hygiene, Medical Faculty, Technische Universität Dresden, Fetscherstraße 74, 01307 Dresden, Germany; 4 Present Address: SLK-Kliniken Heilbronn, 74080 Heilbronn, Germany

**Keywords:** Cystic fibrosis, *Pseudomonas aeruginosa*, Multidrug resistance, Broth microdilution, Azithromycin, SCFM

## Abstract

Long-term treatment with azithromycin is a therapeutic option in Cystic Fibrosis (CF) patients chronically infected with *P. aeruginosa*. It was recently shown that azithromycin has direct antimicrobial activity when *P. aeruginosa* isolates are tested in Roswell Park Memorial Institute medium supplemented with fetal calf serum (RPMI 1640/FCS) by broth microdilution. We now investigated whether (i) azithromycin might also be active against multidrug resistant (MDR) *P. aeruginosa* isolated from CF patients and (ii) how in vitro sensitivity assays perform in synthetic cystic fibrosis sputum medium (SCFM), a medium that mimics the particular CF airway environment. In 17 (59%) out of 29 MDR *P. aeruginosa* CF isolates MICs for azithromycin ranged between 0.25 and 8 μg/ml and 12 isolates (41%) showed a MIC ≥512 μg/ml when measured in RPMI/FCS. In contrast, MICs were ≥ 256 μg/ml for all *P. aeruginosa* MDR isolates when tested in either SCFM or in conventional cation-adjusted Mueller Hinton Broth. High MIC values observed in CF adapted medium SCFM for both PAO1 and MDR *P. aeruginosa* CF isolates, as opposed to findings in RPMI, argue against routine azithromycin MIC testing of CF isolates.

## Background

Cystic fibrosis (CF) is a chronic disorder caused by autosomal-recessive mutations in the cystic fibrosis transmembrane conductance regulator (CFTR) gene. Airway infections with *Pseudomonas aeruginosa* (*P. aeruginosa*) are common in CF patients. They are associated with a decline in lung function, thereby contributing to increased mortality [1]. Current therapeutic strategies aim at eradicating initial or first infection with *P. aeruginosa*. When eradication fails, chronic infection can develop and then therapy tries to suppress *P. aeruginosa* load [2, 3].

Yet, antibiotic targeting of *P. aeruginosa* can be challenging as approximately 20% of *P. aeruginosa* positive patients have been reported by the North American CF registry to carry multidrug resistant (MDR) strains, as defined by resistance to all routinely tested antibiotics in two or more of the following classes: β-lactams, fluoroquinolones and aminoglycosides [4]. In these patients, inhaled antibiotics such as tobramycin, colistin and aztreonam have been suggested as therapy of choice due to the high concentrations that can be achieved upon local delivery [5].

Moreover, in patients chronically infected with *P. aeruginosa*, long-term treatment with macrolides, especially azithromycin, is an accepted therapeutic option and is progressively becoming standard of care [5–9]. The positive effect of azithromycin on clinically relevant end points, including increase in FEV1 (forced expiratory volume in 1 s, a parameter used to assess lung function) and lower risk of pulmonary exacerbations [9], has primarily been attributed to anti-inflammatory and anti-virulence activities of azithromycin. Indeed, sub-inhibitory concentrations of azithromycin have been demonstrated to impair motility, quorum sensing and virulence factor expression including protease activity in *P. aeruginosa* [10].

Although traditionally *P. aeruginosa* is considered to be intrinsically resistant to macrolides, recent data indicate that macrolides may possess an in vitro antimicrobial activity against *P. aeruginosa* depending on the medium used for susceptibility testing by broth microdilution (BMD) [11, 12]. Thus, minimal inhibitory concentrations (MICs) of azithromycin were significantly lower in Roswell Park Memorial Institute medium (RPMI 1640), a medium commonly used for culturing eukaryotic cells, in bronchoalveolar lavage fluid or in cation-adjusted Mueller Hinton Broth (CA-MHB) supplemented with serum as compared to MICs measured conventionally in CA-MHB alone [11, 12]. Therefore, it was suggested that MIC assessment of azithromycin in *P. aeruginosa* CF isolates using RPMI 1640 could be implemented as routine diagnostic measurement in microbiology laboratories [12].

However, it has not been studied whether azithromycin also exhibits antimicrobial activity against MDR *P. aeruginosa*, especially in the context of CF disease. Of note, previously used test media like RPMI1640/FCS do not truly reflect the physiological airway environment observed in CF patients which might affect the interpretation of antibiotic susceptibility. In the present study, we therefore set out (i) to investigate the in vitro efficacy of azithromycin in MDR *P. aeruginosa* isolates derived from respiratory specimen of CF patients by (ii) using different media for BMD. Test media were CA-MHB, RPMI 1640 and synthetic cystic fibrosis sputum medium (SCFM), a medium mimicking the nutritional composition of CF sputum that was suggested to reflect physiological conditions [13].

## Methods

### Study population and routine microbiological analysis of samples

The study was done as a retrospective study on *P. aeruginosa* strains stored from CF patients who received in- or out-patient medical care at the University Hospital between January 2013 and December 2016. From those patients *P. aeruginosa* strains that were tested in the routine microbiology laboratory had been stored in skim milk at − 80 °C. The surveillance of multi-resistant organisms is performed in concordance to the German Infection Protection Act. The local ethics advisory board of the Heidelberg University Hospital was consulted prior to study begin for conformity with the current regulations (S-474/2018). Strain selection is described in the results section in detail. Identification at the species level of isolates cultured from respiratory samples was performed with MALDI-TOF (Bruker) and/or VITEK®2 (Biomerieux). Routine susceptibility testing was performed on the VITEK®2 system for fast growing isolates using the VITEK®2 test card AST-N248 for gram-negative bacteria, while agar diffusion was used for slowly growing isolates in accordance with current German guidelines for microbiological laboratory standards [14]. Evaluation of colistin susceptibility was performed additionally within this study in cryopreserved isolates by BMD in concordance with current EUCAST (European Committee on Antimicrobial Susceptibility Testing) recommendations [15] using the commercially available Micronaut-S MIC strips (Merlin Diagnostics, Germany). All data for antimicrobial susceptibility testing were interpreted according to EUCAST clinical breakpoints.

### Determination of azithromycin MIC by BMD

Azithromycin MICs were determined by BMD in 96-well microtiter plates in a concentration range of 0.125 μg/ml to 1024 μg/ml according to current diagnostic standards [16, 17]. Briefly, *P. aeruginosa* clinical isolates or laboratory control strain PAO1 were grown overnight on Columbia Blood Agar plates at 36 +/− 1 °C and inoculated into CA-MHB, RPMI 1640 with stable glutamine supplemented with 30% fetal calf serum (FCS) or synthetic cystic fibrosis sputum medium (SCFM) [13] at a final concentration of 5 × 10^5^ CFU/ml. Microtiter plates were incubated at 36 +/− 1 °C at ambient atmosphere, according to EUCAST guidelines and in line with Buyck et al. [11] MICs were routinely read at 20 h. For some isolates, incubation was extended to 48 h due insufficient growth at 20 h. MICs were read as the lowest concentration of azithromycin at which visible growth was inhibited. Two isolates failed to grow in SCFM and were therefore excluded from analysis. Reference strain PAO1 has been described previously [18] and was obtained from the Leibniz Institute DSMZ (German Collection of Microorganisms and Cell Lines, #22644).

### Determination of *P. aeruginosa* growth curves

Bacterial growth curves were evaluated by using the Cell Growth Quantifier system (CGQ, Aquila Biolabs). CGQ is a technology for non-invasive real-time monitoring of biomass in shake flasks which is based on the measurement of the amount of light scattered towards a sensor as a function of the current biomass concentration inside the flask. To this end, *P. aeruginosa* was inoculated at a final concentration of 5 × 10^5^ CFU/ml into the indicated culture media, transferred into Erlenmeyer conical flasks and shaken in the dark in 5% CO2, endvolume 10 ml, at 36 +/− 1 °C, 200 rpm. Backscattered light was continuously measured by CGQ over 24 h.

### 23S rRNA mutation detection

PCR amplification and sequencing of domain V of 23S rRNA genes of *P. aeruginosa* isolates, for which mutations have been described, were done as described previously [12] with forward primer: 5′-GGTGCCGGAAGGTTAATTGATG-3′, and reverse primer: 5′-GCAGCCCCTCTCAAATCTCAAAC-3′.

### Statistical analysis

Data were analyzed using the STATA13 software (STATACorp, USA). Statistical analysis of AZM MICs in different test media was performed by non-parametric Kruskal-Wallis test with Dunn’s correction for multiple comparison using GraphPad Prism Software. A *p*-value of < 0.05 was considered statistically significant.

## Results

### Study population

Between January 2013 and December 2016, we received respiratory materials from 930 CF patients. *P. aeruginosa* was identified in 292 patients out of which 49 (=16.8%) carried MDR *P. aeruginosa* (Table 1). MDR was defined according to the rules of the German Commission for Hospital Hygiene and Infection Prevention (KRINKO) as combined resistance to piperacillin, piperacillin/tazobactam, ceftazidime, imipenem, meropenem and ciprofloxacin. This definition is also in line with the one of the North American CF registry [4, 5]. Random isolates from approximately two-thirds (*n* = 30/49) of these MDR *P. aeruginosa* positive patients had been cryopreserved in skim milk at − 80 °C. As two isolates were not cultivable and as one sample contained two different MDR isolates, 29 isolates from 28 patients were finally included in the present study. All patients were classified as chronic *P. aeruginosa* carriers with the exception of one patient with an intermittent carriage status [19]. Most patients in the study cohort were aged between 21 and 40 years. The youngest patient who was tested positive for MDR *P. aeruginosa* was 7 years old. Co-resistance to other antibiotics in MDR *P. aeruginosa* was common: all isolates were resistant to aztreonam, 69% to fosfomycin and resistance to aminoglycosides ranged from 62% (tobramycin) to 93% (gentamicin). Non-susceptibility to colistin was observed in 17% of isolates (Table 1). For tobramycin and colistin, antimicrobial susceptibility testing was interpreted for systemic administration as neither EUCAST nor CLSI (Clinical & Laboratory Standards Institute) provide breakpoint values for local application of these antibiotics via inhalation.

**Table 1:** Basic data on microbiological findings within the study population

	**CF patients**	
	n	
**Total**	930	
***P. aeruginosa*** **pos.**	292	
***P. aeruginosa*** **MDR pos.**^**a**^	49 (=16.8% of *P. aeruginosa* pos.)	
	**MDR** ***P. aeruginosa*,** ^b^	
	n	%
**Age**^**c**^		
0–10	3	11
11–20	2	7
21–30	10	36
31–40	7	25
41–50	2	7
> 51	4	14
**Resistance to** ^**d**^		
Colistin	5	17
Fosfomycin	20	69
Aztreonam	29	100
Gentamicin	27	93
Tobramycin	18	62
Amikacin	25	86
**Carriage status** ^**e**^		
Intermittent	1	4
Chronic	27	96

^a^ defined as combined resistance to piperacillin/tazobactame, ceftazidime, imipenem, meropenem and ciprofloxacin

^b^ *N* = 29 isolates from *N* = 28 patients, i.e. one patient had two morphologically distinct isolates

^c^ median age 27.5 years (range 7 to 70 years); *n* = 28 patients

^d^ categorized as resistant (i.v. application) if MIC interpreted as intermediate or resistant

^e^ chronic: > 50% *P. aeruginosa* positive samples within 12 months; intermittent: < 50% *P. aeruginosa* positive samples within 12 months; negative: > 1 year *P. aeruginosa* negative

### Determination of azithromycin MIC by BMD in MDR *P. aeruginosa* using different test media

**Fig. 1:**
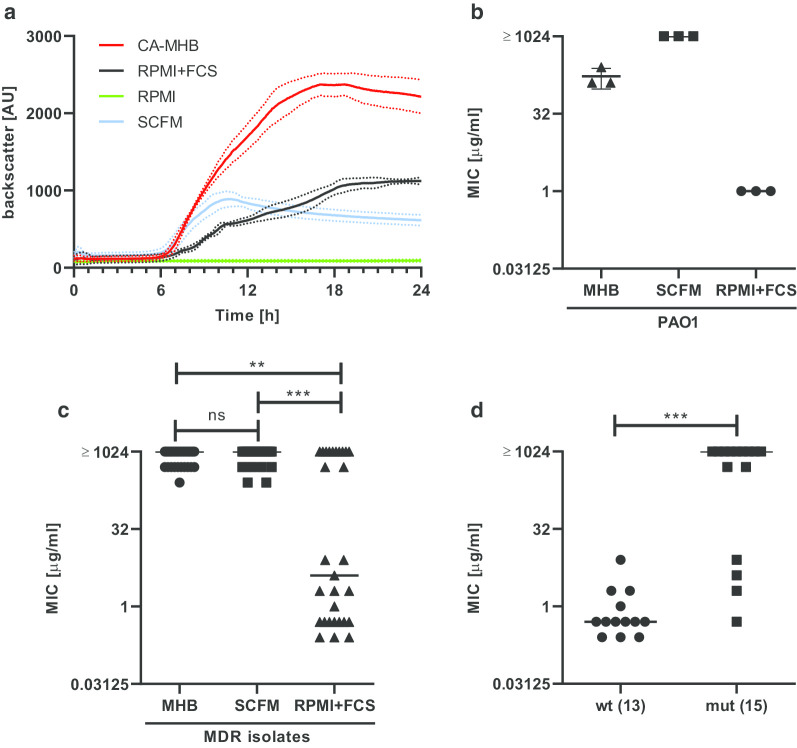
Evaluation of azithromycin MICs against *P. aeruginosa* strain PAO1 and MDR *P. aeruginosa* CF isolates in different media. **a** *P. aeruginosa* strain PAO1was inoculated in the indicated media and increase in bacterial growth was continuously evaluated over 24 h by measuring backscattered light intensity using Cell Growth Quantifier system. CA-MHB: cation-adjusted Mueller Hinton Broth; RPMI: Roswell Park Memorial Institute 1640 medium; FCS: fetal calf serum; SCFM: synthetic cystic fibrosis sputum medium. Data indicate mean (solid lines) +/− SD (dotted lines) from three independent experiments. **b**, **c** MICs of azithromycin were determined by broth microdilution in *P. aeruginosa* strain PAO1 (**b**) and in multidrug resistant (MDR) clinical *P. aeruginosa* isolates derived from cystic fibrosis patients (**c**) using CA-MHB, SCFM and RPMI 1640 supplemented with 30% FCS as test medium. Bars indicate mean +/− SD from three independent experiments (**b**) or median values (**c**). For MDR isolates, *n* = 29 for CA-MHB and RPMI/FCS and *n* = 27 for SCFM (Two isolates failed to grow in SCFM and were therefore excluded from analysis). Statistical analysis was performed using a Kruskal-Wallis test and Dunn’s test for multiple comparison. (**) *p* < 0.01; (***) *p* < 0.001; ns: not significant (**d**) MIC distribution in RPMI/FCS for strains that showed either wildtype (wt) or mutant (mut) sequence in 23S rRNA (pos. 2045, 2046 and 2598)

Previous studies suggested that the medium used for BMD critically influences the in vitro susceptibility of *P. aeruginosa* towards azithromycin [11, 12]. Yet it remains unclear if azithromycin exerts direct antimicrobial effects also in MDR *P. aeruginosa* isolates and in CF adapted test medium. We therefore determined azithromycin MICs in (i) MDR *P. aeruginosa* CF clinical isolates using (ii) different media for BMD including CA-MHB (medium commonly used for BMD), RPMI 1640 (eukaryotic cell culture medium used by [11, 12]) and SCFM (medium mimicking CF airway milieu). Unlike previously described [11], *P. aeruginosa* reference strain PAO1 failed to grow in RPMI 1640 alone but required the presence of FCS (Fig. 1a). Yet, in line with the data of Buyck et al. [11], azithromycin MICs against PAO1 were 1 μg/ml when measured in RPMI supplemented with 30% FCS and ranged between 128 and 256 μg/ml in CA-MHB in three independent experiments (Fig. 1b). Surprisingly, in SCFM, azithromycin MIC of PAO1 was reproducibly determined with ≥1024 μg/ml and was thus even slightly higher than in CA-MHB (Fig. 1b). Of note, in MDR *P. aeruginosa* clinical isolates derived from CF patients, two distinct populations became evident when azithromycin MICs were assessed in RPMI/FCS: In 17/29 MDR isolates (59%), MIC ranged between 0.25 and 8 μg/ml whereas 12/29 MDR isolates (41%) had a MIC of ≥512 μg/ml (Fig. 1c). However, MICs were ≥ 256 μg/ml for all MDR isolates when measured either in CF adapted medium SCFM or conventional CA-MHB (Fig. 1c).

To study whether the mechanism of resistance against azithromycin might affect the differing susceptibility in RPMI/FCS, mutations at three positions within 23S rRNA (2045, 2046 and 2598 in *Pseudomonas aeruginosa* PAO1 corresponding to position 2058, 2059 and 2611 in the 23S subunit of *Escherichia coli*) known to be associated with azithromycin resistance [12, 20] were analyzed. We found mutations at one of the three positions in 23S rRNA in 53.6% of the isolates (15 out of 28 isolates that could be sequenced) whereas 46.4% (*N* = 13) showed wildtype sequence at those positions. Four types of mutations were detected: A2045G, A2046C, A2046G, A2598T. Of note, when analyzing the MICs in RPMI/FCS for wildype vs. mutant strains, it became obvious that 11 from 15 mutant strains had MICs ≥512 μg/ml, whereas for wildtype strains all but one had MIC < 8 μg/ml.

## Discussion

Several clinical studies have validated the beneficial effects of long-term treatment with azithromycin in CF patients chronically infected with *Pseudomonas aeruginosa* (*P. aeruginosa*) and its usage has progressively entered clinical guidelines [5–9]. The efficiency of azithromycin has been attributed to its anti-inflammatory and anti-virulence properties including e.g. inhibition of motility, quorum sensing and protease activity [9, 10, 21, 22]. Although *P. aeruginosa* is considered naturally resistant to macrolides, in vitro susceptibility was previously demonstrated upon testing in alternative media including eukaryotic cell medium RPMI 1640 (supplemented or not with FCS) or serum-supplemented CA-MHB, suggesting that macrolides might additionally exert direct antimicrobial activity on *P. aeruginosa* [10, 11]. The differences observed in phenotypic susceptibility to azithromycin depending on the test medium have been ascribed to increased outer-membrane permeability and decreased expression of efflux pumps in the presence of RPMI 1640 or serum, leading to enhanced azithromycin accumulation inside the bacteria [11]. The authors therefore proposed that azithromycin MIC testing of *P. aeruginosa* CF isolates in RPMI 1640 could routinely be included in microbiological diagnostics [11].

Extending previous findings, we demonstrate here that in 17 out of 29 (59%) MDR *P. aeruginosa* CF isolates, MIC values were low when tested in RPMI supplemented with FCS, ranging from 0.25–8 μg/ml. In contrast, in vitro resistance with high MICs to azithromycin even in RPMI/FCS as found in 12 out of 29 MDR isolates in the present study could be explained by mutations in the 23S rRNA which are frequently detected in CF isolates. Indeed, Mustafa et al. observed mutations in domain V of 23S rRNA in 43% of CF *P. aeruginosa* isolates while mutations were absent in 48 tested strains derived from patients suffering from hospital acquired pneumonia [12]. Thus, testing in RPMI/FCS might be an option to identify *P. aeruginosa* resistance caused by 23S rRNA mutation.

However, although RPMI and CA-MHB supplemented with FCS have been suggested to more closely resemble the eukaryotic environment and therefore to constitute the better test medium, these media do not necessarily reflect the particular milieu in the airways of CF patients. It was suggested that the physiological situation of CF airways might be better mimicked by SCFM which imitates the specific nutritional composition and ion concentrations of CF sputum and is therefore a less rich medium compared to RPMI [13]. We therefore evaluated susceptibility of MDR *P. aeruginosa* in this medium. Of note, azithromycin MICs were consistently ≥256 μg/ml in SCFM in all *P. aeruginosa* clinical isolates as well as in reference strain PAO1, arguing against a direct antimicrobial effect of azithromycin in the airways of CF patients. Macrolides are protonated in acidic environments going along with reduced activity. SCFM was used with a pH of 6.8, which might interfere with activity, yet, a slightly acidic pH in airways of CF patients is well documented in the literature [13]. As a conclusion, our data therefore do not support routine azithromycin MIC assessment in CF clinical isolates using RPMI/FCS, as proposed previously [12]. This study shows that for CF isolates and macrolides in vitro testing is associated with a high level of uncertainty. SCFM, sputum adapted medium, might be more appropriate for antimicrobial susceptibility testing than conventional broth. This notion is also supported by a recent publication of Diaz Iglesias et al who investigated antibiotic susceptibility, biofilm formation and metabolic activity using different media [23].

Our results do not substantiate a direct antimicrobial effect of azithromycin on *P. aeruginosa* when tested in SCFM, a medium that represents the CF environment. Even though we could not detect an antibacterial effect of azithromycin on *P. aeruginosa* we could replicate the occurrence of a resistance mechanism, specifically the mutation of the 23S rRNA gene, in MDR isolates of CF patients. This might be interpreted as a hint that azithromycin has an effect on *P. aeruginosa* in vivo other than blocking bacterial growth. Indeed it has been demonstrated that azithromycin causes a modulation of protein expression instead of a complete block which would be needed for an antibacterial effect. This effect seems to be pronounced for genes involved in quorum sensing [22, 24, 25]. Since this effect seems to take place at concentration far below the measured MIC and the quorum sensing system is involved in biofilm formation, virulence and immune modulation [26, 27] it is plausible that this drives the development of azithromycin resistance in *P. aeruginosa* in vivo. Since azithromycin does not seem to be effective in CF patients uninfected with *P. aeruginosa* [28] this indicates that azithromycin has indeed an effect on *P. aeruginosa* in vivo. Therefore it would be interesting to study the effect of azithromycin in patients with azithromycin resistant strains compared to patients with non-resistant strains. In this context testing for azithromycin resistance using RPMI might be a useful tool.

In conclusion our data do not support the implementation of azithromycin MIC assessment of *P. aeruginosa* CF isolates in routine microbiological diagnostics as suggested previously [12]. The results warrant further assessment of the in vivo efficacy of azithromycin in the subgroup of MDR *P. aeruginosa* infected CF patients in prospective clinical trial.

## Data Availability

The datasets used and/or analysed during the current study are available from the corresponding author on reasonable request.

## Abbreviations

CF: Cystic fibrosis
*P. aeruginosa*: *Pseudomonas aeruginosa*
CFU: Colony forming units
SCFM: Synthetic Cystic Fibrosis Sputum Medium
CA-MBH: Cation adjusted Mueller Hinton Broth
RPMI: Roswell Park Memorial Institute
FCS: Fetal calf serum
BMD: Broth microdilution
MIC: Minimal inhibitory concentration
MDR: Multi-drug resistance
LIS: Laboratory information system
CGQ: Cell Growth Quantifier
